# Autophagy in thyroid cancer: stage-dependent switch, mutation-specific regulation, and therapeutic targeting

**DOI:** 10.3389/or.2026.1873752

**Published:** 2026-07-08

**Authors:** Lihua Fang, Chaowen Wu, Huimin Sun, Jie Ning

**Affiliations:** 1 Department of Endocrinology, Shenzhen Longhua District Central Hospital, Shenzhen, Guangdong, China; 2 Department of Medical Laboratory, Shenzhen Longhua District Central Hospital, Shenzhen, Guangdong, China

**Keywords:** autophagy, BRAF V600E, hydroxychloroquine, stage-dependent switch, therapeutic resistance, thyroid cancer

## Abstract

Autophagy plays context-dependent roles in thyroid cancer, but whether its dual nature represents a reversible balance or an irreversible stage-dependent switch has remained unclear. This review synthesizes evidence across papillary, follicular, medullary, and anaplastic thyroid carcinoma subtypes and reveals that the transition from tumor-suppressive to tumor-promoting autophagy is unidirectional and driven by disease progression, not a simultaneous equilibrium. The major driver mutations BRAF V600E, RAS, and RET regulate autophagy through distinct mechanisms, demanding subtype-specific therapeutic strategies rather than a uniform approach. Importantly, autophagy is an adaptive resistance mechanism rather than a primary driver, as no thyroid cancer is driven by mutations in autophagy genes. Consequently, autophagy inhibitors will not work as monotherapy but may sensitize tumors to standard treatments that create therapeutic stress. Current clinical translation remains stalled by poor inhibitor specificity, lack of validated biomarkers, and unknown treatment timing. We conclude that the field should move from descriptive studies to biomarker-enriched, hypothesis-driven trials, prioritizing anaplastic thyroid carcinoma for hydroxychloroquine-TKI combinations while standardizing assays for p62 and LC3B to enable patient selection.

## 1 Introduction

Thyroid cancer (TC) is the most common endocrine malignancy, with rising incidence worldwide over recent decades (1). While most cases, particularly papillary thyroid carcinoma (PTC), have favorable prognoses, a substantial subset progresses to advanced, metastatic, or therapy-resistant disease. Anaplastic thyroid carcinoma (ATC) and medullary thyroid carcinoma (MTC), though less frequent, remain clinically challenging due to their aggressiveness and limited treatment options (2). A central feature of TC biology is the frequent dysregulation of the MAPK and PI3K/AKT/mTOR pathways, driven by mutations in BRAF (especially BRAF V600E), RAS, and RET rearrangements. These alterations promote tumorigenesis and progression, but they also create vulnerabilities. Notably, resistance to targeted therapies such as BRAF inhibitors and tyrosine kinase inhibitors (TKIs) often arises through compensatory survival mechanisms, among which autophagy has emerged as a key player.

Autophagy is a lysosome-dependent catabolic process that maintains cellular homeostasis by degrading damaged organelles and misfolded proteins (3). In cancer, autophagy plays a context-dependent, dual role. It suppresses early tumor initiation by eliminating potentially oncogenic materials, yet in established tumors, it promotes survival by helping cells adapt to metabolic stress and therapeutic insults. This duality is particularly relevant in TC, where autophagy has been implicated in proliferation, invasion, metastasis, and resistance to radioactive iodine (RAI) and TKIs. Despite growing interest in targeting autophagy for cancer therapy, several critical questions remain unanswered in TC. How do subtype-specific molecular alterations (BRAF, RAS, RET) differentially regulate autophagic flux? Under what conditions does autophagy switch from tumor-suppressive to tumor-promoting? Can autophagy modulation be effectively integrated into existing clinical regimens, including RAI, TKIs, and immunotherapy?

This review systematically examines the molecular mechanisms linking autophagy to key driver mutations in TC, evaluates its dual role across disease stages, analyzes its contribution to therapy resistance, and discusses emerging strategies for autophagy-based precision oncology. By bridging bench-side discoveries and clinical translation, we aim to provide a roadmap for more effective, personalized treatment of thyroid cancer.

## 2 Molecular landscape and autophagy regulation in thyroid cancer subtypes

Thyroid cancer comprises four major histological subtypes, papillary, follicular, medullary, and anaplastic, each defined by distinct molecular alterations that also shape how autophagy is regulated. Understanding this subtype-specific landscape is prerequisite to targeting autophagy therapeutically. Papillary thyroid carcinoma, the most common subtype, frequently harbors BRAF V600E mutations or RET/PTC rearrangements, both leading to constitutive MAPK pathway activation. In PTC cells, BRAF V600E exerts a complex, context-dependent effect on autophagy. On one hand, sustained MAPK signaling upregulates anti-apoptotic BCL-2, which binds Beclin-1 and inhibits autophagy initiation. On the other hand, BRAF inhibition (e.g., with vemurafenib) paradoxically induces ER stress and activates the transcription factors TFEB and TFE3, driving autophagic flux that promotes resistance (4, 5). Hypoxia further complicates this picture. HIF-1α transcriptionally activates TERT, which suppresses mTOR and induces autophagy, supporting PTC cell survival under hypoxic stress (6). Thus, in PTC, autophagy is not simply “on” or “off” but is dynamically tuned by oncogenic signaling, therapeutic pressure, and microenvironmental stress.

Follicular thyroid carcinoma is molecularly distinct, characterized by RAS mutations and PAX8/PPARγ rearrangements that frequently dysregulate the PI3K/AKT pathway, a major negative regulator of autophagy. PI3K/AKT/mTOR activation suppresses autophagic flux, but therapeutic agents that inhibit this pathway can reverse that suppression. For example, the VEGFR2 inhibitor, piperlongumine, exerts antitumor effects in FTC by inhibiting PI3K/AKT/mTOR, thereby increasing both autophagy and apoptosis (7). Similarly, piperlongumine induces ROS-mediated autophagy and apoptosis in FTC cells via the ROS/Akt pathway. The key point is that in FTC, the PI3K/AKT/mTOR axis acts as a rheostat for autophagy, and its dysregulation creates both a vulnerability and a potential therapeutic target. Medullary thyroid carcinoma arises from parafollicular C cells and is driven primarily by RET mutations. Direct mechanistic studies of autophagy in MTC remain limited, but several observations suggest specialized roles. Immune checkpoint molecules such as TIGIT are frequently expressed in MTC cells, hinting at an immunomodulatory aspect that may intersect with autophagic pathways (8). TROP-2, a transmembrane glycoprotein, is also highly expressed in MTC, raising the possibility that antibody-drug conjugates could modulate autophagic flux in this subtype (9). Whether RET signaling directly regulates autophagy in MTC, and whether that regulation contributes to secretory function or cell survival, awaits further investigation.

Anaplastic thyroid carcinoma is the most aggressive and lethal subtype, frequently accumulating mutations in TP53 and TERT promoter alongside concurrent activation of MAPK and PI3K/AKT pathways. Autophagy is robustly induced in ATC, where it serves as a survival mechanism under harsh microenvironmental conditions. Berberine induces ROS generation and activates both autophagy and apoptosis in ATC cells through the PI3K/AKT/mTOR pathway (10). Homoharringtonine (HHT) impairs lysosomal function and autophagosome-lysosome fusion, leading to cytotoxicity via TFEB-dependent inhibition of lysosomal biogenesis (11). Expression of autophagy-related molecules such as LC3B and p62 is altered in ATC and correlates with aggressive behavior and genomic instability (12). Moreover, mitochondrial quality control via mitophagy is disrupted in ATC. The tumor suppressors MIEAP and ATG5 play critical roles in modulating mitochondrial accumulation and tumor development (13). In contrast to PTC or FTC, where autophagy can be either protective or suppressive depending on context, ATC exhibits a consistent pattern, high autophagic activity supports tumor survival and therapy resistance, making autophagy inhibition a particularly attractive strategy in this subtype.

Core machinery and its subtype-specific configurations. The core autophagic apparatus, the ULK1 complex (initiation), the class III PI3K complex (nucleation), and the ATG12-ATG5-ATG16L1/LC3 systems (elongation), are conserved across all TC subtypes (14, 15). What differs is how upstream regulators, particularly mTORC1 and AMPK, are regulated. Under nutrient-rich conditions, active mTORC1 phosphorylates ULK1 and suppresses autophagy. Nutrient deprivation, energy stress, or growth factor withdrawal inhibits mTORC1 and releases ULK1. AMPK, activated by low energy, directly phosphorylates and activates ULK1 while inhibiting mTORC1 (16). In TC, subtype-specific aberrations in the MAPK and PI3K/AKT pathways alter the setpoints of this regulatory network. Hypoxic PTC cells exhibit mTOR inhibition via the HIF-1α/TERT axis (6). Apatinib and nilotinib induce autophagy by inhibiting PI3K/Akt/mTOR (17). In oncocytic thyroid tumors, defective mitophagy due to MIEAP downregulation leads to accumulation of abnormal mitochondria and increased ROS production (18). Even transcription factors TFEB and TFE3, master regulators of lysosomal biogenesis, are overexpressed in PTC and correlate with enhanced autophagy-lysosome activity that promotes invasion and metastasis (19).

**FIGURE 1:**
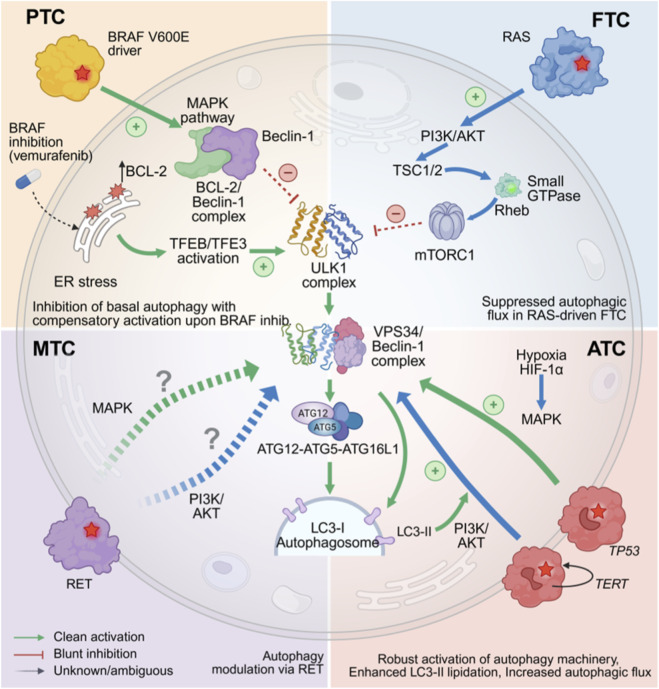
Subtype-specific autophagy regulation by driver mutations in thyroid cancer. BRAF V600E suppresses basal autophagy but induces compensatory autophagy upon BRAF inhibition. RAS mutations suppress autophagy via PI3K/AKT/mTOR activation, while ATC exhibits robust pro-survival autophagy driven by combined MAPK/PI3K/AKT activation and TP53/TERT mutations. Created with BioRender.com.

Rather than treating autophagy as a uniform process, therefore, the molecular landscape of TC demands a subtype-specific view. PTC is characterized by dynamic, context-tuned autophagy influenced by BRAF, hypoxia, and therapy. FTC by PI3K/AKT-mediated suppression that can be therapeutically reversed. MTC by poorly understood but potentially distinct RET- and immune-related autophagic roles and ATC by consistently high, pro-survival autophagy that renders it susceptible to autophagy inhibition. These differences form the foundation for the dual role of autophagy explored in the following sections. The subtype-specific regulators of autophagy in thyroid cancer are summarized in Figure 1; Table 1.

**TABLE 1:** Key autophagy-regulating molecules and their functional consequences in thyroid cancer subtypes.

Subtype	Molecule/Pathway	Regulation of autophagy	Functional consequence	Key references
PTC	BRAF V600E → MAPK → BCL-2	Inhibition (baseline)	Reduced autophagic cell death	(4, 5)
PTC	HIF-1α/TERT → mTOR suppression	Activation (under hypoxia)	Hypoxic adaptation, progression	(6)
PTC	HMGB1 (upon vemurafenib)	Activation (compensatory)	BRAF inhibitor resistance	(5)
PTC	SQSTM1/p62 → AKT/AMPK/mTOR	Bidirectional	Promotes growth; triggers autophagy	(20)
PTC	DUSP4 → JNK-BCL2-Beclin1	Inhibition	Suppresses autophagic cell death	(21)
PTC	Ref-1 → AMPK activation	Activation (oxidative stress)	Overloads autophagic flux, senescence	(22)
PTC	TRIB2 → Wnt/β-catenin → ERK/AKT	Activation (resistance)	EMT, invasiveness, vemurafenib resistance	(23)
FTC	RAS → PI3K/AKT/mTOR	Inhibition (baseline)	Maintains metabolic homeostasis	(24, 25)
FTC	USP7 → NEK2/ATG5	Activation (upon sorafenib)	Sorafenib resistance	(26)
FTC	Piperlongumine → ROS/Akt	Induction	Apoptosis and autophagy	(7)
MTC	RET → MAPK and PI3K/AKT	Underexplored	Unknown; may affect secretory function	(8, 27, 28)
ATC	TP53/TERT + MAPK/PI3K/AKT	Strong activation	Metabolic adaptation, survival	(10–13)
ATC	Berberine → PI3K/AKT/mTOR	Induction	Apoptosis and autophagy	(10)
ATC	Homoharringtonine (HHT)	Inhibition (lysosomal dysfunction)	Cytotoxicity via TFEB	(11)
ATC	MIEAP/ATG5 (mitophagy)	Suppression (defective)	Tumor progression	(13)
ATC	NIS → LIR motif → autophagic degradation	Activation (autophagy-mediated)	RAI resistance, impaired iodine uptake	(29–31)
All subtypes	LC3B, p62 expression	Variable (correlative)	Associated with aggressiveness	(12)

Abbreviations: PTC, papillary thyroid carcinoma; FTC, follicular thyroid carcinoma; MTC, medullary thyroid carcinoma; ATC, anaplastic thyroid carcinoma; ROS, reactive oxygen species; TERT, telomerase reverse transcriptase; HIF-1α, hypoxia-inducible factor 1α; HMGB1, high mobility group box 1; USP7, ubiquitin-specific protease 7; NEK2, NIMA-related kinase 2; ATG5, autophagy-related gene 5; TFEB, transcription factor EB; MIEAP, mitochondria-eating protein; NIS, sodium-iodide symporter; LIR, LC3-interacting region.

## 3 The dual role of autophagy in thyroid cancer initiation and progression

Autophagy contributes to thyroid cancer in two opposing ways depending on disease stage, genetic context, and microenvironmental conditions. In early carcinogenesis, autophagy primarily suppresses tumor development by maintaining genomic integrity and eliminating damaged organelles. In established tumors, particularly advanced or dedifferentiated subtypes like ATC, cancer cells co-opt autophagy as a survival mechanism to withstand metabolic stress and therapeutic insults.

### 3.1 Tumor-suppressive autophagy in early carcinogenesis

During initial stages of thyroid transformation, moderate autophagic activity prevents malignant progression through several interconnected mechanisms. Clearance of damaged mitochondria and misfolded proteins limits oxidative stress and DNA damage, reducing the likelihood of oncogenic mutations. Consistent with this protective role, downregulation of autophagy-related genes (ATGs) or impairment of autophagic flux promotes malignant transformation and invasiveness in preclinical models. Specific molecular evidence supports this tumor-suppressive function. The autophagy adaptor SQSTM1/p62 mediates both autophagy and apoptosis in PTC cells via the AMPK/AKT/mTOR pathway. Elevated p62 expression correlates with enhanced cell growth, while p62 deficiency inhibits proliferation and induces apoptosis (20). DUSP4 inhibits JNK-BCL2-Beclin1 signaling, thereby suppressing autophagic cell death in PTC. Paradoxically, DUSP4 knockdown enhances JNK phosphorylation, promotes Beclin1 release from BCL2, and induces autophagy-mediated cell death, ultimately suppressing tumor growth (21).

Mitochondrial quality control provides another layer of tumor suppression. The mitophagy-related protein MIEAP and ATG5 function as tumor suppressors in BRAF V600E-positive thyroid cancer models. Knockout of either MIEAP or ATG5 accelerates cancer development, indicating that both canonical and noncanonical mitophagy pathways prevent malignant progression by maintaining mitochondrial homeostasis (13). In thyroid oncocytic tumors, defective mitophagy leads to accumulation of abnormal mitochondria and increased ROS production, with MIEAP downregulated in these cancers (18). MicroRNAs also contribute to tumor-suppressive autophagy. miR-203a-3p inhibits PTC progression by suppressing MAP3K1 and activating autophagy (32). Silencing the long non-coding RNA DLX6-AS1 enhances autophagy and apoptosis through upregulation of miR-193b-3p and downregulation of HOXA1 (33). Collectively, these findings indicate that intact autophagic machinery acts as a barrier against thyroid carcinogenesis. Autophagy defects, whether through genetic alterations, signaling dysregulation, or epigenetic modifications, promote malignant transformation.

### 3.2 Tumor-promoting autophagy in advanced and treatment-resistant disease

Once thyroid tumors are established, particularly in aggressive or dedifferentiated forms, autophagy switches to a pro-survival function. Cancer cells exploit autophagic degradation to recycle intracellular components, thereby sustaining metabolic demands under nutrient deprivation, hypoxia, and therapeutic stress. In ATC and aggressive PTC harboring BRAF V600E mutations, autophagy is markedly induced under hypoxic stress through the HIF-1α/TERT axis, which inhibits mTOR and activates autophagic flux (6). In BRAF V600E-mutant cells, autophagy sustains mitochondrial respiration by regulating fatty acid oxidation, inhibiting autophagy sensitizes these cells to vemurafenib (34). More broadly, autophagy provides rapidly proliferating TCcells with essential metabolic substrates, amino acids and fatty acids, supporting biosynthesis and energy production in the nutrient-poor tumor microenvironment.

Autophagy also directly mediates therapy resistance. In BRAF-mutant PTC cells, HMGB1 promotes vemurafenib resistance by driving excessive autophagy, which reduces apoptosis and enhances cell viability. Targeting HMGB1 or inhibiting autophagy reverses this resistance (5). In FTC, USP7 promotes sorafenib resistance by enhancing NEK2/ATG5-mediated autophagy. USP7 knockdown reduces autophagic flux and sensitizes cells to sorafenib (26). RAF inhibitors combined with MEK blockers often encounter resistance mediated by autophagy activation, suggesting that co-targeting autophagy may enhance efficacy (35). LINC00162 modulates MAPK signaling and apoptosis in TC cells. Its silencing enhances sorafenib sensitivity, partly through autophagy regulation (36). MicroRNAs further influence drug resistance by regulating autophagy-related genes (37). In ATC, where autophagy is most prominently upregulated, it contributes to both aggressiveness and treatment resistance. Capsaicin inhibits ATC stemness by inducing autophagy-lysosome-mediated degradation of OCT4A (38). Combinations of autophagy inducers and immune checkpoint inhibitors show synergistic effects in promoting autophagic cell death in ATC cells (39). Autophagy also sustains mitochondrial function and energy metabolism in ATC. Its inhibition disrupts these processes, sensitizing cells to targeted therapies (40).

**FIGURE 2:**
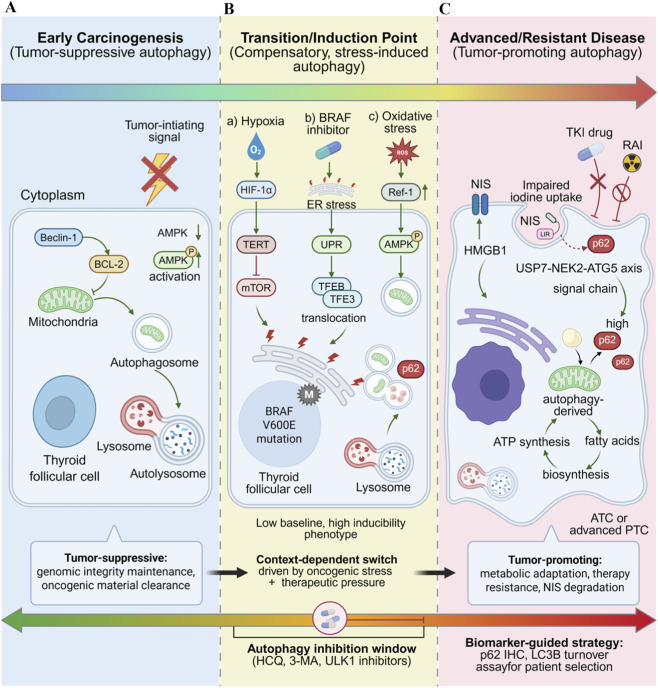
Stage-dependent switch of autophagy in thyroid cancer. **(A)** Early carcinogenesis: autophagy suppresses tumors via Beclin-1 release, AMPK activation, and mitochondrial clearance. **(B)** Transition point: BRAF V600E establishes a “low-baseline, high-inducibility” phenotype—hypoxia (HIF-1α/TERT→mTOR↓), therapeutic stress (ER stress→TFEB/TFE3), and oxidative stress (Ref-1→AMPK) converge to drive cytoprotective autophagic flux. **(C)** Advanced disease: autophagy sustains metabolic adaptation (HMGB1, USP7-NEK2-ATG5 axis) and NIS degradation (LIR motif-mediated), conferring RAI and TKI resistance. Autophagy inhibition (HCQ) offers a therapeutic window for biomarker-selected patients. Created with BioRender.com.

The shift from tumor-suppressive to tumor-promoting autophagy in TC is not binary but gradual, influenced by the balance between stress intensity and autophagic capacity. Low-level, transient autophagy removes damaged components and suppresses transformation. High-level, sustained autophagy, often driven by hypoxia, therapy, or oncogenic stress, becomes a dependency for cancer cell survival. This duality has direct therapeutic implications, restoring or enhancing autophagy may prevent early lesions, whereas inhibiting autophagy in advanced disease may overcome resistance and improve treatment outcomes (Figure 2).

## 4 Crosstalk between driver mutations and autophagy in thyroid cancer

The major driver mutations in thyroid cancer, BRAF V600E, RAS, and RET,do not merely promote proliferation and survival. They also rewire autophagic activity. Understanding this crosstalk is critical because it determines how tumor cells respond to stress and therapy, and it reveals context-specific vulnerabilities for therapeutic intervention.

### 4.1 BRAF V600E: dual and therapy-linked regulation of autophagy

BRAF V600E, the most prevalent driver in PTC, constitutively activates the MAPK pathway. Its effect on autophagy is not fixed but shifts depending on therapeutic pressure and cellular stress. Under baseline conditions, sustained MAPK signaling upregulates anti-apoptotic BCL-2, which binds and sequesters Beclin-1, thereby inhibiting autophagy initiation and creating a low-baseline autophagy state (24). This tonic suppression, however, establishes a “low-baseline, high-inducibility” phenotype: the capacity for stress-induced autophagy is markedly enhanced because convergent stress pathways, HIF-1α/TERT-mediated mTOR suppression (6), BRAF inhibitor-triggered ER stress/TFEB-TFE3 activation (5), and Ref-1-driven AMPK activation (22), collectively relieve BCL-2-mediated Beclin-1 sequestration and drive cytoprotective autophagic flux (41). Thus, the apparent contradiction between baseline suppression and stress-induced activation reflects a context-dependent, bidirectional regulatory mechanism rather than a true paradox (42, 43).

Paradoxically, when BRAF V600E-mutant cells are treated with BRAF inhibitors such as vemurafenib. ER stress is induced, triggering the unfolded protein response and activating the transcription factors TFEB and TFE3. The result is a surge in autophagic flux that buffers drug-induced proteotoxicity and apoptosis (5). This therapy-induced, cytoprotective autophagy is a major mechanism of acquired resistance. Resistant cells show upregulation of TRIB2, regulated by Wnt/β-catenin signaling, which enhances ERK/AKT activation, EMT, and invasiveness. TRIB2 knockdown sensitizes resistant cells back to vemurafenib (23). Oxidative stress induced by BRAF activation upregulates Ref-1, conferring intrinsic resistance. Pharmacological inhibition of Ref-1 combined with vemurafenib overloads autophagic flux via excessive AMPK activation, leading to senescence and cell death (22).

### 4.2 RAS mutations: autophagy suppression via PI3K/AKT/mTOR activation

RAS mutations, particularly prevalent in FTC, activate both MAPK and PI3K/AKT pathways (25). The PI3K/AKT arm is the dominant regulator of autophagy in this context. Activated AKT phosphorylates and inhibits TSC1/2, a negative regulator of mTORC1, resulting in sustained mTORC1 activation and subsequent suppression of autophagic flux (44). This contrasts with BRAF V600E, where baseline autophagy is often low but can be therapeutically induced. In RAS-mutant FTC, autophagy is tonically suppressed by oncogenic PI3K/AKT signaling (27).

However, when therapeutic agents, such as AKT or PI3K inhibitors, are applied, this suppression can be relieved, and autophagic flux may rebound as a compensatory survival mechanism. Tumor cells can engage feedback loops that reactivate autophagy, enhancing survival and resistance (45). This adaptive response underscores the plasticity of FTC cells and explains why single-agent targeting of the PI3K/AKT pathway often yields only transient effects.

Molecular profiling reveals that RAS mutations frequently coexist with other alterations (46), such as TERT promoter mutations, which are associated with more aggressive behavior (47). DICER1 mutations define a distinct molecular subset of FTC, particularly in pediatric cases (48, 49). While RAS mutations generally correlate with low-to-intermediate grade phenotypes, their effect on autophagy, suppression via PI3K/AKT/mTOR, remains a consistent driver of tumorigenesis and a potential therapeutic node. Combining PI3K/AKT/mTOR inhibitors with autophagy inducers (to push beyond a toxic threshold) or with autophagy inhibitors (to block compensatory rebound) represents two opposing but potentially valid strategies that require context-specific evaluation.

### 4.3 RET rearrangements and mutations: underexplored but convergent signaling

RET rearrangements (in PTC) and activating mutations (in MTC) drive signaling through both MAPK and PI3K/AKT pathways, similar to RAS, but with distinct downstream consequences for autophagy. In principle, RET-driven PI3K/AKT activation should suppress autophagy via mTORC1. However, the available evidence suggests a more complex pattern. In MTC, where direct mechanistic studies of autophagy are limited, several observations hint at specialized roles. Constitutive RET activation may modulate autophagic flux through mechanisms that affect not only cell survival but also the secretory phenotype of parafollicular C cells (28). Immune checkpoint molecules such as TIGIT are frequently expressed in MTC, suggesting potential links between RET signaling, autophagy, and immune modulation (8).

Therapeutically, multi-kinase inhibitors (MKIs) such as vandetanib and cabozantinib target RET which are approved for advanced MTC, but resistance frequently develops. One emerging mechanism is the induction of compensatory autophagy, which allows tumor cells to survive despite kinase inhibition. Combining MKIs with autophagy inhibitors (e.g., chloroquine) enhances cytotoxic effects in preclinical models (50). Selective RET inhibitors (selpercatinib, pralsetinib) have shown remarkable clinical responses in RET fusion-positive PTC and RET-mutant MTC (51). However, resistance to these selective inhibitors, through on-target RET mutations or bypass signaling, has been reported, and autophagy may play a role in this resistance as well (52). Different RET fusion partners (e.g., CCDC6-RET, NCOA4-RET) correlate with distinct clinicopathological features and may influence autophagic activity and therapeutic responses (53).

The three major driver mutations in TC regulate autophagy through overlapping yet distinct mechanisms. BRAF V600E exerts a bidirectional effect: low baseline autophagy due to BCL-2-mediated sequestration of Beclin-1, but strong cytoprotective autophagy induction under therapeutic stress. RAS mutations tonically suppress autophagy through PI3K/AKT/mTOR activation, with rebound autophagy upon pathway inhibition. RET signaling likely combines elements of both, depending on cellular context and specific fusion partners. Recognizing these differences not merely of theoretical interest. It directly informs whether a given patient’s tumor might benefit from autophagy inhibition (to block therapy-induced survival) or, paradoxically, from autophagy enhancement (to push stressed cells over a death threshold).

## 5 Autophagy-mediated resistance to conventional therapies

Resistance to standard treatments, radioactive iodine (RAI) and tyrosine kinase inhibitors (TKIs), is a major clinical obstacle in advanced thyroid cancer. Across both modalities, autophagy has emerged as a central adaptive mechanism that allows tumor cells to survive therapeutic stress. Understanding how autophagy is engaged and how to block it offers a rational strategy to restore treatment sensitivity.

### 5.1 Radioactive iodine resistance: autophagy and NIS dysregulation

RAI therapy remains the standard adjuvant and salvage treatment for differentiated thyroid carcinoma (DTC), relying on sodium-iodide symporter (NIS)-mediated iodine uptake into thyroid cells. Resistance (RAIR-DTC) develops in a subset of patients, particularly those with metastatic or recurrent disease, and correlates with poor prognosis (54, 55). Autophagy contributes to RAI resistance through two distinct molecular mechanisms involving NIS dysregulation (56). The first mechanism involves autophagic degradation of NIS protein. NIS contains putative LC3-interacting region motifs that enable recognition by the autophagic machinery (29, 30). Under oncogenic stress driven by BRAF V600E or TERT promoter mutations, upregulated autophagic flux sequesters NIS into autophagosomes, leading to lysosomal degradation and reduced total NIS protein levels. The second mechanism involves disruption of NIS trafficking to the plasma membrane (54, 57). Autophagy-related proteins, particularly ATG5 and ATG7, compete with NIS trafficking adaptors such as the AP-2 clathrin adaptor complex for binding to endosomal sorting machinery. This competition diverts NIS away from the plasma membrane and toward autophagic degradation (31). Additionally, autophagy-induced lysosomal dysfunction alters endosomal pH, impairing post-translational modifications including glycosylation and phosphorylation that are required for NIS membrane insertion and functional iodide transport (58). Pharmacological autophagy inhibition using chloroquine or 3-methyladenine stabilizes NIS protein, restores its plasma membrane localization, and partially restores, validating autophagy as a rational co-target for reversing RAI resistance (59).

Molecular alterations that drive TC progression also intersect with this autophagic resistance. BRAF and TERT mutations, which are overrepresented in RAIR-DTC, both downregulate NIS expression and promote autophagy-mediated survival (45). Conversely, pharmacological inhibition of autophagy can restore RAI sensitivity. Autophagy blockade enhances RAI-induced apoptosis and partially recovers iodine uptake by stabilizing NIS expression and promoting its localization to the plasma membrane (60).

Redifferentiation therapies aimed at re-inducing NIS expression have shown variable clinical success (61). Metabolic interventions such as ketogenic diets also enhance NIS expression and RAI uptake, possibly through effects on autophagy (60). For patients who remain RAI-refractory despite these strategies, multikinase inhibitors (sorafenib, lenvatinib) are the current standard of care (62, 63). The key point is that autophagy sits at the intersection of NIS regulation and radiation survival, making it a rational co-target for reversing RAI resistance.

### 5.2 Tyrosine kinase inhibitor resistance: autophagy as a metabolic lifeline

TKIs such as lenvatinib and sorafenib are cornerstones of therapy for advanced RAIR-DTC, MTC, and ATC. Acquired resistance, however, is nearly universal. Among multiple resistance mechanisms, including alternative pathway activation, on-target mutations, and phenotypic switching, autophagy has emerged has emerged as a particularly tractable target. Tumor cells adapt to TKI-induced stress by upregulating autophagic flux. Degradation and recycling of intracellular components provide metabolic substrates that maintain ATP levels and biosynthesis, effectively creating a “metabolic restart” that allows cells to persist despite ongoing kinase inhibition. In FTC, USP7 promotes sorafenib resistance by enhancing NEK2/ATG5-mediated autophagy. USP7 stabilizes NEK2 via deubiquitination, upregulating ATG5 and facilitating autophagic flux. USP7 knockdown reduces autophagy and sensitizes cells to sorafenib (26).

Clinically, lenvatinib demonstrates superior progression-free survival (PFS) compared to sorafenib in RAIR-DTC (median PFS approximately 13.2 months for sorafenib, up to 35.3 months for lenvatinib), but both agents eventually fail (64, 65). Rechallenge with lenvatinib after progression on sorafenib has shown tumor resensitization in some cases, suggesting that resistance mechanisms, including autophagy, may be reversible (66). Prolonged TKI exposure also induces phenotypic changes, EMT, activation of pro-survival pathways, and upregulation of oncogenes such as CEACAM and NUPR1, that further engage autophagic support (67). Preclinical evidence strongly supports combining autophagy inhibitors with TKIs. Hydroxychloroquine (HCQ) blocks autophagosome-lysosome fusion and significantly enhances antitumor efficacy while delaying resistance in animal models (68). In ATC, where TKI monotherapy yields limited benefit (median OS 4.8–6.4 months), synergistic induction of autophagic cell death has been observed when TKIs are combined with histone deacetylase inhibitors and immune checkpoint inhibitors (69).

Clinical management of TKI resistance currently relies on careful patient selection, timing of therapy initiation, and monitoring of biomarkers such as neutrophil-to-lymphocyte ratio (NLR) (70, 71). Adverse events, hypertension, proteinuria, hand-foot syndrome, fatigue, often necessitate dose modifications that can compromise efficacy (72). Emerging targeted therapies, including selective RET inhibitors (selpercatinib, pralsetinib) and BRAF/MEK inhibitors (dabrafenib plus trametinib), offer improved specificity and potentially reduced toxicity (73, 74). Regardless of the specific TKI, the pattern is consistent, autophagy is engaged as a survival response, and its inhibition enhances cell death. RAI and TKIs target different vulnerabilities, iodine uptake and kinase signaling, respectively, but both create cellular stress that autophagy helps to mitigate. In RAI resistance, autophagy degrades NIS and buffers radiation damage. In TKI resistance, autophagy provides metabolic support that allows cells to outlast drug exposure. In both settings, the therapeutic implication is the same: adding an autophagy inhibitor does not merely add toxicity. It removes an adaptive shield that would otherwise limit the efficacy of the primary treatment.

## 6 Therapeutic targeting of autophagy in thyroid cancer

Given the dual role of autophagy, tumor-suppressive in early disease, tumor-promoting in advanced and treatment-resistant disease, therapeutic targeting must be context-dependent. Two broad strategies have emerged, autophagy inhibition to block pro-survival functions in established tumors, and autophagy induction to eliminate damaged cells or restore sensitivity in specific contexts.

### 6.1 Autophagy inhibition in advanced and resistant disease

In advanced TC, particularly RAIR-DTC, ATC, and tumors resistant to TKIs, autophagy functions as a cytoprotective mechanism that buffers therapeutic stress. Inhibiting this survival pathway is therefore a rational adjunct to standard treatments. Hydroxychloroquine (HCQ) is the most clinically advanced autophagy inhibitor. It accumulates in lysosomes, raises intra-lysosomal pH, and blocks autophagosome-lysosome fusion, thereby preventing degradation of autophagic cargo. In preclinical models of PTC, combining HCQ with lenvatinib enhances apoptosis and anti-angiogenic effects (68). HCQ has been evaluated in clinical trials for solid tumors, though specific data in TC remain limited. Its main limitations are modest potency and off-target effects, including retinal toxicity.

Early-stage autophagy inhibitors offer greater specificity but are largely confined to preclinical research. 3-methyladenine (3-MA) targets the class III PI3K/VPS34 complex and blocks autophagy initiation. It sensitizes BRAF V600E-mutant TC cells to vemurafenib by preventing the compensatory autophagic surge that otherwise limits drug efficacy (5). ULK1 inhibitors, which block the most upstream step in autophagy initiation, have shown activity in preclinical models but have not yet entered clinical testing for TC.

The therapeutic logic for autophagy inhibition is straightforward: if a tumor upregulates autophagy to survive chemotherapy, TKI, or RAI, then blocking that upregulation should increase cell death. Experimental evidence supports this logic across multiple TC models. Autophagy inhibition reduces cancer stem cell viability, migration, and invasion (75). It sensitizes ATC cells to doxorubicin and berberine (10), and restores NIS function and enhances radioiodine uptake (76). The key question is not whether autophagy inhibition works in principle, but how to identify which patients' tumors are autophagy-dependent and how to combine inhibitors with primary therapies for maximal effect.

Early-stage autophagy inhibitors such as 3-methyladenine and ULK1 inhibitors remain stalled in preclinical phases due to multiple interconnected barriers (77). A fundamental limitation is their poor specificity and consequent off-target toxicity. 3-methyladenine inhibits class III phosphatidylinositol 3-kinase, which governs not only autophagy but also endosomal trafficking, receptor signaling, and immune cell function, so systemic administration causes broad cellular dysfunction including immunosuppression and metabolic disruption (78). Unlike hydroxychloroquine, which produces measurable lysosomal pH elevation as a pharmacodynamic readout, 3-methyladenine and ULK1 inhibitors lack clinically trackable endpoints such as LC3-II turnover or p62 accumulation to confirm target engagement, making dose optimization in phase I trials extremely difficult (79). Another critical obstacle is compensatory pathway activation, blocking autophagy at the initiation stage triggers adaptive survival responses through mTOR hyperactivation, Nrf2 antioxidant signaling, and alternative degradation routes including the proteasome and chaperone-mediated autophagy, which collectively undermine monotherapy efficacy (80). Furthermore, ULK1 plays an essential role in neuronal homeostasis and metabolic adaptation, so systemic inhibition risks neurotoxicity and metabolic crisis, creating a narrow therapeutic window (81). Without tumor-specific delivery strategies such as nanoparticle encapsulation or thyroid-targeted prodrugs, these agents cannot achieve therapeutic concentrations in tumors while sparing normal tissues. These cumulative barriers explain why 3-methyladenine and ULK1 inhibitors remain research tools, whereas hydroxychloroquine, despite its modest potency and off-target effects, remains the only clinically accessible autophagy inhibitor (82).

### 6.2 Autophagy induction: a context-limited strategy

Autophagy induction is conceptually attractive in two scenarios, preventing malignant transformation in premalignant lesions, and clearing therapy-induced cellular debris that might otherwise fuel inflammation or tumor recurrence. In practice, however, autophagy induction as a therapeutic strategy in established TC carries significant risks, because once a tumor is formed, the same induction may inadvertently support survival. Metformin, an antidiabetic drug that activates AMPK and inhibits mTOR, promotes autophagic flux. In PTC models, metformin inhibits tumor growth and metastasis, partly through autophagy activation (83). Whether this effect is mediated by autophagy or by other AMPK-dependent mechanisms remains unclear. Other agents reported to induce autophagy in TC include piperlongumine (via ROS-mediated PI3K/Akt inhibition in FTC) (7) and Sho-saiko-to (via PI3K-AKT pathway modulation) (84).

The cautionary note is critical. Radiotherapy induces autophagy in TC cells, but that autophagic response counteracts apoptosis; therefore, inhibiting autophagy rather than enhancing it increases radiation-induced cell death (85). Similarly, while moderate autophagy may clear damaged organelles and suppress transformation, once a tumor is established, further autophagy induction risks providing the metabolic support that enables progression and resistance. For these reasons, autophagy inducers remain predominantly research tools rather than ready for clinical use therapeutics. Their potential lies in well-defined contexts: prevention in high-risk individuals, or timed pulses to clear residual disease after cytoreductive therapy, but these applications require rigorous preclinical validation and careful patient selection.

The opposing strategies of autophagy inhibition and induction are not contradictory. They reflect the stage-dependent biology of autophagy in TC. In early or premalignant lesions where autophagic capacity is intact but suboptimal, boosting autophagy may restore homeostasis and prevent transformation. In advanced, treatment-resistant tumors where autophagy is hijacked as a survival mechanism, inhibiting it removes a critical shield. The challenge for clinical translation is not choosing one strategy over the other, but developing biomarkers that reliably tell which phase a given patient’s tumor is in. Preclinical evidence and clinical bottlenecks of autophagy-targeted strategies are summarized in Table 2.

**TABLE 2:** Autophagy-targeted therapeutic strategies in thyroid cancer: preclinical evidence and clinical translation bottlenecks.

Strategy	Agent/Method	Mechanism of action	Preclinical/Clinical evidence	Major bottleneck	Key references
Autophagy inhibition (advanced disease)	Hydroxychloroquine (HCQ)	Blocks autophagosome-lysosome fusion	Synergistic with lenvatinib in PTC models	Retinal toxicity, modest potency	(68)
Autophagy inhibition (initiation phase)	3-Methyladenine (3-MA)	Inhibits VPS34 (class III PI3K)	Sensitizes BRAF V600E-mutant PTC to vemurafenib	Poor specificity; off-target toxicity; no validated PD biomarker; compensatory pathway activation	(5, 77, 78, 80)
Autophagy inhibition (novel)	ULK1 inhibitors	Blocks ULK1 kinase activity	Effective in preclinical cancer models	Not yet tested in thyroid cancer; narrow therapeutic window; neurotoxicity risk; compensatory mTOR/Nrf2 activation	(79, 81)
Autophagy inhibition (novel)	VPS34 inhibitors	Targets nucleation complex	Preclinical activity in solid tumors	Not yet tested in thyroid cancer	(82)
Combination with TKI	HCQ + lenvatinib/sorafenib	Blocks adaptive survival autophagy	Enhances apoptosis, reduces resistance in models	No thyroid cancer-specific phase III trial	(25, 63, 68)
Combination with immunotherapy	HCQ + anti-PD-1/PD-L1	Enhances tumor immunogenicity, promotes ICD	Synergistic cytotoxicity in ATC cell lines	Lack of thyroid cancer-specific clinical data	(31, 86)
Neoadjuvant modulation	Short-course HCQ ± TKI/ICI	Cytoreduction, primes for adjuvant therapy	Window-of-opportunity for locally advanced tumors	No thyroid cancer-specific trial; biomarker selection needed	(87–89)
Autophagy induction (prevention/early disease)	Metformin	Activates AMPK, inhibits mTOR	Suppresses PTC growth (partially autophagy-dependent)	Risk of promoting survival in established tumors	(83)
Autophagy induction	Piperlongumine	ROS-mediated PI3K/Akt inhibition	Induces apoptosis and autophagy in FTC	Preclinical only; context-dependent	(7)
Autophagy induction	Sho-saiko-to	PI3K-AKT pathway modulation	Promotes apoptosis and redifferentiation	Preclinical only; mechanism unclear	(84)
Biomarker-guided strategy	p62 immunohistochemistry	Identifies autophagy-dependent tumors	Correlated with aggressiveness in retrospective studies	Not prospectively validated	(12, 20)
Biomarker-guided strategy	LC3B turnover (LC3-II/I ratio)	Measures autophagic flux	Candidate for patient selection	No standardized assay	(19)
Biomarker-guided strategy	PD-L1 + TIL density + p62/LC3B panel	Stratifies autophagy dependency and immune context	Retrospective correlation	Not prospectively validated; requires multi-center standardization	(86, 90)

Abbreviations: PTC, papillary thyroid carcinoma; FTC, follicular thyroid carcinoma; ATC, anaplastic thyroid carcinoma; TKI, tyrosine kinase inhibitor; ICI, immune checkpoint inhibitor; HCQ, hydroxychloroquine; ICD, immunogenic cell death; ROS, reactive oxygen species; ULK1, unc-51, like autophagy activating kinase 1; VPS34, vacuolar protein sorting 34; AMPK, AMP-activated protein kinase; mTOR, mammalian target of rapamycin; IHC, immunohistochemistry; TIL, tumor-infiltrating lymphocyte; PD, pharmacodynamic.

Beyond inhibition, controlled autophagy induction represents an emerging strategy in immuno-oncology, particularly for anaplastic thyroid carcinoma where immune checkpoint blockade alone shows limited efficacy (86). Autophagy induction promotes immunogenic cell death, characterized by the release of damage-associated molecular patterns including ATP, HMGB1, and calreticulin (86). These molecular signals activate dendritic cells and facilitate cytotoxic T lymphocyte recruitment, potentially enhancing anti-PD-1 or anti-PD-L1 therapy (91). Preclinical evidence in anaplastic thyroid carcinoma models demonstrates that combining autophagy inducers such as mTOR inhibitors or AMPK with activators immune checkpoint inhibitors increases tumor antigenicity and promotes immunogenic cell death. However, this strategy requires precise control, as excessive autophagy may stabilize PD-L1 and promote immune evasion (92), while insufficient induction fails to trigger the desired cell death response. Biomarker-guided patient selection through measurement of baseline autophagic flux, PD-L1 expression, and tumor-infiltrating lymphocyte density is essential to identify suitable candidates for this combinatorial approach (90).

## 7 Autophagy, the tumor microenvironment, and immunotherapy

Autophagy modulates not only tumor cell–intrinsic survival but also the tumor immune microenvironment (TIME). In thyroid cancer, this crosstalk remains incompletely understood, but emerging evidence suggests that autophagy influences immune cell infiltration, antigen presentation, and the expression of immune checkpoint molecules. These interactions have direct implications for combining autophagy modulators with immunotherapies, which currently show limited efficacy in thyroid neoplasms.

### 7.1 Autophagy shapes immune recognition and suppression in the TIME

The effect of autophagy on anti-tumor immunity is bidirectional. On one hand, autophagic degradation of intracellular proteins generates peptides for MHC loading, potentially enhancing antigen presentation and T-cell recognition. Autophagy also facilitates immunogenic cell death (ICD), a form of cell death that releases damage-associated molecular patterns (DAMPs) such as ATP, HMGB1, and calreticulin, which recruit and activate dendritic cells and cytotoxic T lymphocytes. On the other hand, autophagy promotes the secretion of immunosuppressive cytokines and stabilizes immune checkpoint molecules, including PD-L1.

In thyroid cancer, direct evidence for these mechanisms is limited but growing. Autophagy-mediated regulation of PD-L1 has been documented in several solid tumors, and preliminary data in TC suggest that inhibiting autophagy decreases PD-L1 levels, enhancing T cell–mediated cytotoxicity. Conversely, autophagy induction may stabilize PD-L1, promoting immune evasion. Autophagy also influences the balance of immune cell subsets, regulatory T-cell (Tregs), myeloid-derived suppressor cells (MDSCs), and tumor-associated macrophages (TAMs), within the TIME. Studies in macrophages from non-thyroid models show that impaired autophagy leads to lipid accumulation and inflammation, whereas restoring autophagy via ATG14 overexpression promotes Treg populations and attenuates inflammation (93). Whether similar mechanisms operate in thyroid tumor-associated macrophages remains an open question.

### 7.2 Synergy between autophagy modulation and immune checkpoint blockade

Preclinical evidence supports combining autophagy modulators with immune checkpoint inhibitors (ICIs), particularly in aggressive subtypes like ATC. The rationale is twofold, autophagy inhibition may enhance tumor antigen presentation and ICD, thereby overcoming resistance to PD-1/PD-L1 blockade; conversely, controlled autophagy induction may promote ICD and recruit immune effectors. In ATC cell lines, combining PD-L1 blockade with agents that induce autophagic cell death, such as the histone deacetylase inhibitor panobinostat or the TKI sorafenib, demonstrates synergistic cytotoxicity, with enhanced apoptosis and autophagy markers (39). Hydroxychloroquine (HCQ) has been shown to potentiate anti-PD-1/PD-L1 efficacy in preclinical models of other solid tumors by enhancing tumor antigenicity and promoting ICD. Whether HCQ exerts similar effects in TC is not yet established, but the mechanistic logic is compelling.

Clinical data remain the major gap. To date, no large-scale trials have tested the combination of autophagy inhibitors (e.g., HCQ) with ICIs specifically in TC. Early-phase studies are ongoing in other cancer types, and extrapolation to TC, particularly ATC, where ICIs have shown only modest activity, is reasonable but unproven. The primary challenge is not lack of preclinical rationale, but rather the absence of thyroid cancer–specific biomarkers that identify which patients' tumors are both autophagy-dependent and ICI-responsive.

The intersection of autophagy and tumor immunity in TC is understudied compared to other malignancies. Most existing data are derived from non-thyroid models or from *in vitro* TC cell lines without immune components. Key unanswered questions include: Does autophagic flux in TC cells correlate with PD-L1 expression in patient samples? Does autophagy inhibition *in vivo* increase tumor-infiltrating lymphocyte density or activity? Can baseline autophagy status predict response to ICI therapy? Addressing these questions will require dedicated preclinical models (immune-competent mouse models of TC, patient-derived organoids co-cultured with autologous immune cells) and correlative studies embedded in future clinical trials.

## 8 Emerging tools for autophagy research in thyroid cancer

Advances in preclinical models and molecular imaging are transforming how autophagy is studied in thyroid cancer. Patient-derived organoids and xenografts preserve the genetic and histological features of primary tumors, enabling mechanistic studies and drug testing. Meanwhile, non-invasive imaging probes allow real-time monitoring of autophagic flux, potentially guiding patient selection and treatment timing. Together, these tools bridge the gap between basic discovery and clinical application.

### 8.1 Organoid and patient-derived xenograft (PDX) models

Traditional thyroid cancer cell lines, grown in two dimensions and lacking stromal components, poorly recapitulate the heterogeneity and microenvironment of human tumors. Organoid and PDX models address these limitations. Organoids are three-dimensional cultures derived from patient tumor tissue. They retain cellular heterogeneity, genetic architecture, and, to some extent, the hierarchical organization of the original tumor. For autophagy research, organoids enable dynamic monitoring of autophagic flux under controlled conditions, nutrient withdrawal, hypoxia, or drug treatment, while preserving the cellular diversity that influences autophagic responses (94).

PDX models are generated by implanting patient tumor tissue into immunodeficient mice. They maintain histological and genetic integrity over serial passages and have shown high concordance with patient responses to therapy (95). In thyroid cancer, PDX models have been used to profile genomic alterations, including TERT promoter and TP53 mutations, that correlate with aggressive phenotypes and may influence autophagic activity. They also support testing of novel autophagy modulators in combination with targeted therapies such as lenvatinib and provide a platform to study how microenvironmental factors (e.g., hypoxia) regulate autophagy *in vivo* (96).

The limitation of both systems is the absence of a fully functional immune system, which precludes study of autophagy’s role in tumor-immune crosstalk. Immune-humanized PDX models or syngeneic mouse models of TC are needed to address that gap.

### 8.2 Molecular imaging of autophagy activity

Measuring autophagic flux in real time within living tumors is technically challenging but clinically valuable. Static measurements (e.g., LC3-II protein levels by immunohistochemistry) cannot distinguish between high initiation and blocked degradation. Molecular imaging probes offer a dynamic alternative. The most widely used experimental tool is the tandem fluorescent-tagged LC3 reporter (mCherry-GFP-LC3). GFP fluorescence is quenched in the acidic environment of autolysosomes, while mCherry is not. Thus, yellow (merged) signals indicate autophagosomes, while red-only signals indicate autolysosomes, allowing flux to be quantified (97). This system is primarily used in cell lines and xenografts, not in patients. Lysosome-targeted probes represent a more clinically translatable approach. Fluorescent probes that accumulate in lysosomes can visualize lysosomal dynamics and autophagic degradation. Given that lysosomal dysfunction is implicated in TC progression (98), such probes could potentially serve as functional readouts for patient stratification, though this remains investigational.

Nuclear medicine approaches (e.g., radiolabeled autophagy tracers) have been explored in other diseases but lack specificity for autophagic flux and have not been validated in TC (99). For clinical translation, the immediate priority is not developing new probes but validating existing ones, likely starting with *ex vivo* patient samples or intraoperative imaging, before moving to *in vivo* applications. Organoid/PDX models ask “what happens to autophagy in this patient’s tumor under these conditions?” Molecular imaging asks “what is autophagy doing right now in the living system?” Used together, they enable a iterative loop, discovery in models, biomarker development, testing in imaging studies, and refinement back in models. For thyroid cancer, where autophagy's role shifts dramatically by stage and subtype, such tools are not luxuries, they are prerequisites for precision targeting.

## 9 Clinical translation: bottlenecks and priorities

Despite robust preclinical evidence linking autophagy to thyroid cancer progression and therapeutic resistance, clinical translation remains stalled. The first bottleneck is the poor specificity and toxicity of available inhibitors. Hydroxychloroquine (HCQ), the only clinically accessible autophagy inhibitor, blocks lysosomal acidification broadly, causing dose-limiting retinal toxicity and gastrointestinal side effects (68). More selective agents targeting ULK1 or VPS34 exist preclinically but have not entered thyroid cancer trials (34). Addressing this requires prioritizing ATC and therapy-resistant PTC for preclinical testing of HCQ-TKI combinations using patient-derived organoid and PDX models (95), with pharmacodynamic endpoints that confirm autophagic blockade *in situ* rather than relying solely on tumor shrinkage. This limitation is particularly acute for combination with immune checkpoint inhibitors, where preclinical synergy has been demonstrated in ATC models but no thyroid-specific clinical trial has been initiated.

The second bottleneck is the lack of validated biomarkers to identify autophagy-dependent tumors. Autophagic flux is not uniformly elevated across thyroid cancers, nor is it constant within a single tumor over time. Candidate markers such as p62 accumulation by immunohistochemistry and the LC3B turnover ratio have been reported in correlative studies (19, 20), but none has been prospectively validated or standardized across laboratories. Without such biomarkers, trials enrolling unselected patients will inevitably dilute efficacy signals. The priority is therefore to standardize assays for p62 and LC3B and test their predictive value in retrospectively annotated cohorts, then use them to enrich enrollment in prospective trials.

The third bottleneck concerns the optimal timing and sequencing of autophagy inhibition relative to primary therapy. Should an autophagy inhibitor be given before, during, or after TKI or RAI therapy? Continuously or in pulses? Preclinical evidence conflicts: in BRAF V600E-mutant PTC, autophagy is induced as a compensatory response to vemurafenib, and concurrent inhibition sensitizes cells (5), but in other contexts, baseline suppression might be detrimental. Systematic studies of treatment scheduling in immunocompetent, genetically defined mouse models are lacking. The priority is to design biomarker-enriched, adaptive phase II trials that incorporate serial tumor biopsies or, if validated, molecular imaging to confirm target engagement (97), rather than continuing unselected, all-comer studies that are likely to fail regardless of biologic rationale.

**FIGURE 3:**
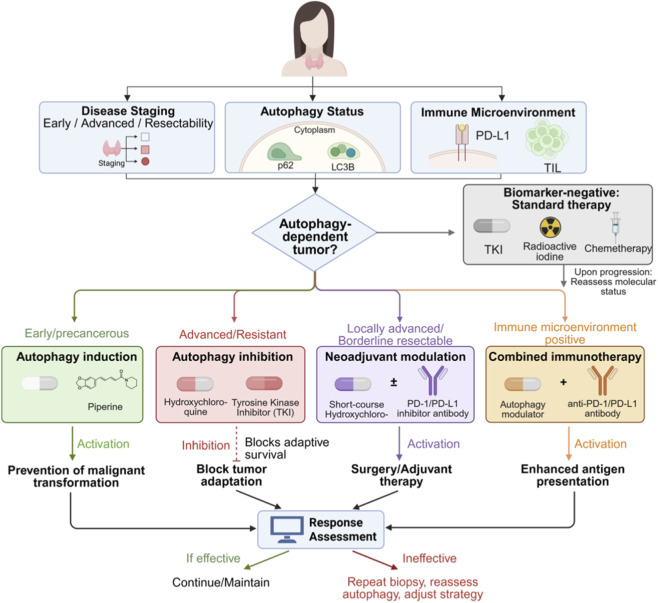
Algorithm for autophagy-targeted therapy in thyroid cancer. Patients are stratified by disease stage, autophagy status (p62, LC3B), and immune microenvironment (PD-L1, TIL). Biomarker-negative tumors receive standard therapy (TKI, RAI, chemotherapy) with reassessment at progression. For autophagy-dependent tumors, four strategies are applied: (1) induction (metformin, piperlongumine) for early lesions; (2) inhibition (HCQ + TKI) for advanced/resistant disease; (3) neoadjuvant modulation (HCQ ± ICI) for locally advanced tumors; and (4) combined immunotherapy (autophagy modulator + anti-PD-1/PD-L1) for immune-positive tumors. Response is assessed at 8–12 weeks; ineffective cases undergo repeat biopsy for strategy adjustment. Created with BioRender.com.

Yet despite this clear rationale, the field currently suffers from too many descriptive studies and too few interventional ones of this kind. The science of autophagy inhibition in thyroid cancer is ready for translation. The clinical infrastructure is not. Closing this gap will require a decisive shift-from cataloging associations to testing hypotheses in biomarker-enriched, mechanistically guided trials that incorporate serial biopsies or molecular imaging to confirm target engagement (Figure 3).

Neoadjuvant autophagy modulation represents a window-of-opportunity strategy for locally advanced or borderline resectable thyroid cancer (87). By administering autophagy-targeted agents prior to definitive surgery or radioiodine therapy, clinicians can exploit a unique temporal window to reshape tumor biology (88). Short-course autophagy inhibition using hydroxychloroquine may reduce tumor viability and prime residual disease for enhanced sensitivity to subsequent adjuvant radioactive iodine or tyrosine kinase inhibitor therapy (89). Conversely, neoadjuvant autophagy induction combined with immune checkpoint blockade may reconfigure the tumor microenvironment to support anti-tumor immunity (100). The selection between induction and inhibition strategies should be guided by pre-treatment biomarker assessment including p62 accumulation, LC3B turnover, and PD-L1 expression levels. Rigorous prospective trials with serial tumor sampling are essential to validate target engagement and biological response. This neoadjuvant paradigm elevates autophagy modulation from a salvage approach to an integral component of multimodal therapy, offering potential to improve outcomes in aggressive thyroid cancer subtypes (101).

## 10 Conclusion

Autophagy modulation is not a uniform strategy. Inhibition removes the survival shield in advanced, therapy-resistant tumors, while controlled induction may promote immunogenic cell death and enhance antigen presentation, particularly when combined with immune checkpoint blockade. The choice between inhibition and induction depends on disease stage, autophagy dependency status, and therapeutic context. This duality, together with mutation-specific regulation by BRAF V600E, RAS, and RET, precludes any singular therapeutic approach. Autophagy inhibitors are not monotherapy candidates but rather adjuncts designed to block adaptive resistance to tyrosine kinase inhibitors or radioactive iodine. Clinical translation has stalled not because the science is immature, but because the field has prioritized descriptive correlations over interventional hypothesis-testing. Moving forward, biomarker-enriched trials, particularly in anaplastic thyroid carcinoma, must test whether hydroxychloroquine-TKI combinations improve outcomes, while next-generation selective autophagy inhibitors are developed. Descriptive studies of autophagy and driver mutation interactions have reached diminishing returns; the urgent next step is mechanistic, hypothesis-driven translation.
